# Effect of Pretreatment with the NADPH Oxidase Inhibitor Apocynin on the Therapeutic Efficacy of Human Placenta-Derived Mesenchymal Stem Cells in Intracerebral Hemorrhage

**DOI:** 10.3390/ijms19113679

**Published:** 2018-11-21

**Authors:** Saehong Min, Ok Joon Kim, Jinkun Bae, Tae Nyoung Chung

**Affiliations:** 1 Department of Emergency Medicine, CHA University School of Medicine, 59 Yatap-Ro, Bundang-Gu, Seongnam 13496, Korea; shmin87@gmail.com (S.M.); galen97@chamc.co.kr (J.B.); 2 Department of Neurology, CHA University School of Medicine, 59 Yatap-Ro, Bundang-Gu, Seongnam 13496, Korea; okjun77@cha.ac.kr

**Keywords:** mesenchymal stromal cells, intracerebral hemorrhage, prognosis, preconditioning, NADPH oxidase, stem cell therapy

## Abstract

Several studies have demonstrated the beneficial effect of mesenchymal stem cells (MSCs) on intracerebral hemorrhage (ICH). Enhancement of the therapeutic efficacy of MSCs in ICH is necessary, considering the diseases high association with mortality and morbidity. Various preconditioning methods to enhance the beneficial properties of MSCs have been introduced. We suggested apocynin, a well-known nicotinamide adenine dinucleotide phosphate (NADPH) oxidase inhibitor, as a novel preconditioning regimen to enhance the therapeutic efficacy of MSCs in ICH. Rat ICH models were made using bacterial collagenase. 24 h after ICH induction, the rats were randomly divided into apocynin-preconditioned MSC-treated (Apo-MSC), naïve MSC-treated and control groups. Hematoma volume, brain edema, and degenerating neuron count were compared at 48 h after the ICH induction. The expression of tight junction proteins (occludin, zona occludens [ZO]-1) were also compared. Hematoma size, hemispheric enlargement and degenerating neuron count were significantly lower in the Apo-MSC group than in the naïve MSC group (*p* = 0.004, 0.013 and 0.043, respectively), while the expression of occludin was higher (*p* = 0.024). Apocynin treatment enhances the therapeutic efficacy of MSCs in ICH in the acute stage, through the improvement of the beneficial properties of MSCs, such as neuroprotection and the reinforcement of endovascular integrity of cerebral vasculature.

## 1. Introduction

Stem cell therapy is currently one of the most promising strategies for the treatment of various neurodegenerative diseases [1,2,3,4]. Recent research showed that mesenchymal stem cell (MSC) therapy could be an effective therapeutic option for global cerebral ischemia [5,6,7,8]. Studies have also shown the beneficial neuroprotective effects of MSCs on animal intracerebral hemorrhage (ICH) models via the secretion of neurotrophic factors [9,10]. Spontaneous ICH is a fatal disease with a significantly poorer prognosis than ischemic stroke, which is highly associated with acute mortality [11,12,13,14,15]. Some genetic diseases such as Anderson-Fabri disease can also cause ICH [16,17]. Symptomatic treatments are currently considered the only effective therapeutic options for ICH, despite various other therapeutic approaches, such as the administration of neuroprotective agents and exogenous coagulation factors, which have been studied to overcome the extremely poor prognosis of ICH [14,18,19]. A recent preclinical study showed that MSCs could prevent hematoma expansion in the hyperacute stage of ICH and decrease acute mortality by enhancing the endothelial integrity of the cerebral vasculature [20]. Nevertheless, there is a necessity to enhance the efficacy of MSCs for the treatment of ICH, considering its extremely poor prognosis.

Various preconditioning strategies including cultural environment controls, 3D culture, and the addition of chemicals or trophic factors have been tried to optimize the therapeutic efficacy of MSCs by enhancing their survival, engraftment, and paracrine properties [21,22]. Hypoxia preconditioning is one of the most widely investigated strategies, which is thought to improve the expression of pro-survival genes and various trophic factors in MSCs, and even to promote their multipotency [23]. Recent research implied the possibility of apocynin as a preconditioning regimen and showed that apocynin partially reversed the aging process in bone marrow-derived MSCs, promoting their potential for proliferation and osteogenesis [24]. However, this research only evaluated the effect of apocynin treatment on the aging process and differentiation potency of MSCs. Furthermore, apocynin is a well-known NADPH oxidase inhibitor, while hypoxia is known to activate NADPH oxidase [25]. Hence, the effect of apocynin-treated MSCs on cerebral ischemia or ICH should be assessed, including a direct comparison with that of naïve MSCs before the consideration of apocynin as a preconditioning regimen.

We aimed to assess the effect of apocynin-preconditioned MSCs on ICH (including direct comparison with that of naïve MSCs) as a proof-of-concept study. We hypothesized that apocynin-pretreatment would enhance the therapeutic effect of MSCs on hematoma size in the acute stage of ICH and ameliorate neuronal death around the hematoma.

## 2. Results

First, 21 rats were enrolled. Three rats died before the intervention groups assignment (24 h after ICH induction). Finally, 18 rats were included in the histologic analyses (6 in each group). Another 12 rats were enrolled for western blotting (4 each in each group). The experimental groups were as follows: (i) Sham group; (ii) vehicle group: ICH + 500 μl saline; (iii) apocynin-preconditioned human placenta-derived MSCs (hPDMSCs) group (Apo-MSC group): ICH + 1 × 10^6^ apocynin-preconditioned hPDMSCs; (iv) naïve MSC group: ICH + 1 × 10^6^ naïve hPDMSCs. 

### 2.1. Effects on Hematoma Volume and Hemispheric Enlargement

**Figure 1:**
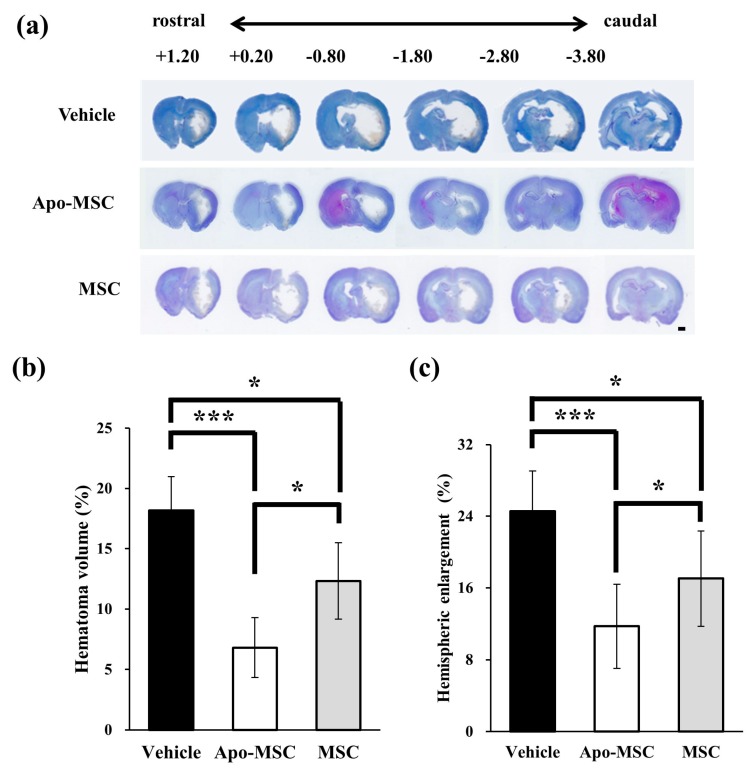
Effect of apocynin-preconditioned human placenta-derived mesenchymal stem cells (Apo-MSCs) and naïve mesenchymal stem cells (MSCs) on hematoma volume and brain edema in the rats at 48 h after the induction of an intracranial hemorrhage (ICH). (**a**) Representative images of cresyl violet staining, depicting the coronal whole-brain section at rostral-caudal levels from +2.04 to −5.52 from the bregma. Unstained area inside brain parenchyma represents hematoma lesion. Scale bar = 1 mm. (**b**) The bar graphs represent the hematoma volume of the Apo-MSCs, naïve MSCs and vehicle treated groups at 48 h after ICH induction. The volume of hematoma is expressed as the proportion of total brain area (%). (**c**) The bar graphs represent hemispheric enlargement of the Apo-MSCs, naïve MSCs and vehicle treated groups at 48 h after ICH induction. The hemispheric enlargement is expressed as the percentage of increase in hemispheric size comparing with that of the contralateral hemisphere. Data are mean + standard deviation (SD). * *p* < 0.05, *** *p* < 0.001.

The hematoma size at 24 h after ICH induction was significantly smaller in both the Apo-MSC group (6.81 ± 2.47%) and the naïve MSC group (12.32 ± 3.17%) compared to the vehicle group (18.17 ± 2.82%, *p* = 0.0001 and 0.04) (Figure 1a,b). The Apo-MSC group also showed more of a reduction effect on hematoma size than the naïve MSC group (*p* = 0.004). To determine the effect on cerebral edema after ICH we measured for changes in hemispheric enlargement. Hemispheric enlargement was significantly smaller in both the Apo-MSC group (11.74 ± 2.20%) and the naïve MSC group (17.05 ± 1.46%) compared to the vehicle group (24.56 ± 3.89%, *p* = 0.003 and 0.02; Figure 1c). Similar to the result for hematoma size, hemispheric enlargement also showed a greatly reduced size in the Apo-MSC group compared to the naïve MSC group (*p* = 0.013). These results indicate that the administration of Apo-MSCs attenuate ICH-induced brain edema formation and hydrocephalus more efficiently than that which was observed in the naïve MSC group.

### 2.2. Effects on Peri-Hematoma Neuronal Death

**Figure 2:**
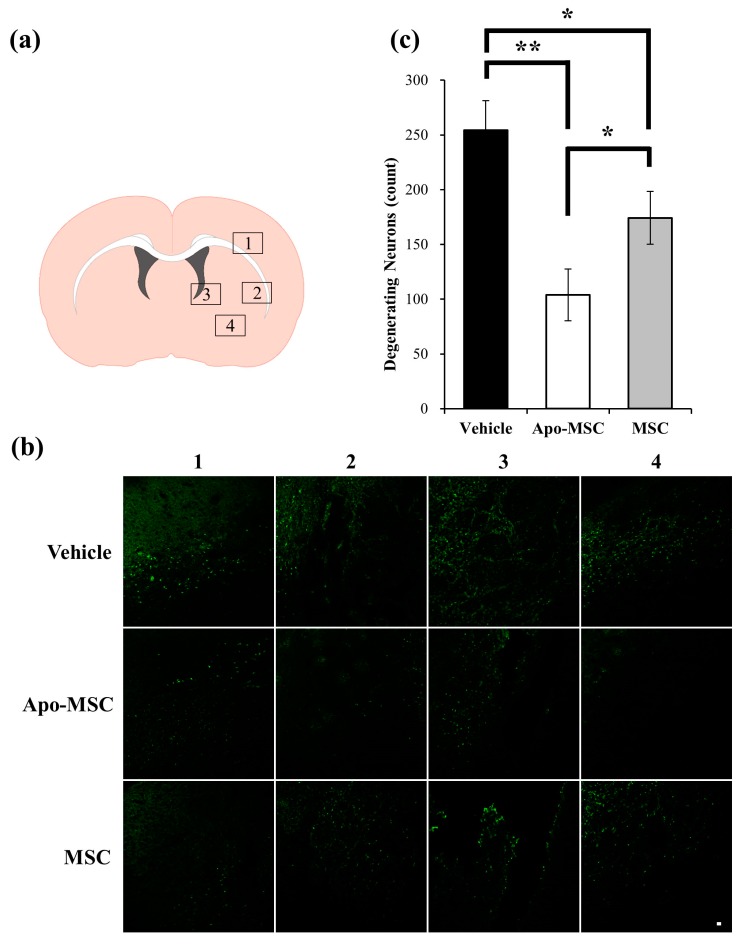
Effect of apocynin-preconditioned human placenta-derived mesenchymal stem cells (Apo-MSCs) and naïve MSCs on the peri-hematoma neuronal death in the rats at 48 h after the induction of an intracranial hemorrhage (ICH). (**a**) The location of core hemorrhagic regions at 0.2 mm from the bregma. Each number represents a region of interest to be analyzed. (**b**) Fluorescence images reveal the degenerating neurons in the peri-hematoma region at 24 h after the induction of an ICH. Degenerating neurons are detected by Fluoro-Jade C (FJC) staining (green). Each number represents a region of interest defined at Figure 2a. Scale bar = 20 μm. (**c**) The bar graphs represent the count of FJC-positive neurons in the peri-hematoma region from the Apo-MSCs, naïve MSCs and vehicle treated groups at 48 h after ICH induction. Data are mean +SD. * *p* < 0.05, ** *p* < 0.01.

To determine the neuroprotective effect of apocynin-treated MSCs on collagenase- induced ICH, we performed Fluoro-Jade C (FJC) staining to detect degenerating neurons. The count of FJC(+) cells in the vehicle-treated group was significantly higher than that in both the Apo-MSC and naïve MSC groups (254.25 ± 26.95, 104.00 ± 23.72, and 174.33 ± 24.24 cells/field, respectively, *p* = 0.0013 and 0.015; Figure 2a–c), while FJC(+) cells were not observed in the contralateral hemisphere. The Apo-MSC group also showed less neuronal death than the naïve MSC group (*p* = 0.043). 

### 2.3. Effects on the Expression of Tight Junction Proteins

**Figure 3:**
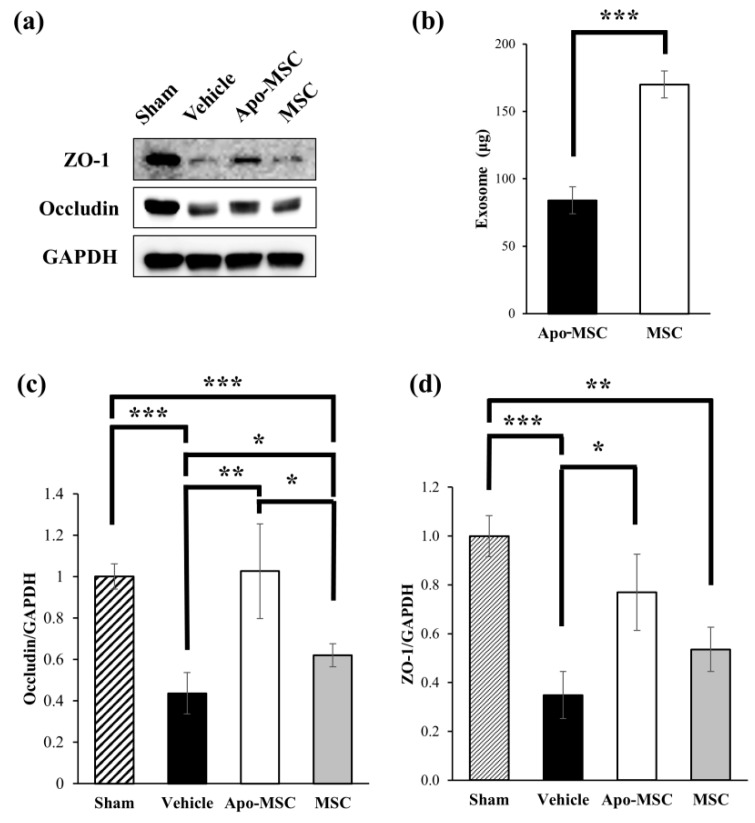
Effect of apocynin-preconditioned human placenta-derived mesenchymal stem cells (Apo-MSCs) and naïve MSCs on the expression of tight junction proteins in the rats at 48 h after the induction of an intracranial hemorrhage (ICH). (**a**) Results of western blotting of occludin and ZO-1 at 48 h after ICH induction. (b) Amount of exosome production. Bar graphs indicate the level of occludin (**c**) and ZO-1 (**d**) expression measured by the densitometric analysis of the bands. Glyceraldehyde 3-phosphate dehydrogenase (GAPDH) was used as a loading control. Data are mean +SD. * *p* < 0.05, ** *p* < 0.01, *** *p* < 0.001.

We investigated the expression of tight junction proteins at 24 h after the administration of apocynin-treated MSCs or naïve MSCs to assess changes in microvascular integrity using western blotting. The level of expression of occludin was significantly higher in the Apo-MSC group and the naïve MSC group than in the vehicle group at 48 h after ICH induction (*p* = 0.003 and 0.023). Moreover, there was a significant difference in the expression level of occludin between the Apo-MSC and naïve MSC groups (*p* = 0.024; Figure 3a,c). The administration of apocynin-treated MSCs induced an increased expression level of occludin compared to the naïve MSC group. The level of expression of ZO-1 was also significantly higher in the Apo-MSC group than in the vehicle group at 48 h after ICH induction (*p* = 0.031). However, there was no significant difference between the vehicle and naïve MSC groups and the Apo-MSC and naïve MSC groups in the expression level of ZO-1 (*p* = 0.116 and 0.140; Figure 3a,d). These results suggest that apocynin-treated MSCs more effectively block the leakage of blood components from ruptured vessels to brain parenchyma after ICH than naïve MSCs. Conclusively, apocynin might induce an enhancement of function in MSCs. As a result, apocynin-treated MSCs reduced the damage of ICH in the acute phase

### 2.4. Effects on the Amount of Exosome Production

We measured and compared the amount of exosomes produced from apocynin-preconditioned MSCs and naïve MSCs to investigate the effect of apocynin preconditioning on exosome production. The amount of exosomes was significantly lower in the culture medium used for apocynin-preconditioned MSCs than that of the naïve MSCs (84.02 ± 10.03 vs 169.96 ± 3.87, *p* < 0.001; Figure 3b).

## 3. Discussion

The results of the present study suggest a proof-of-concept where apocynin-preconditioning can enhance the therapeutic efficacy of MSCs in ICH in the acute stage. We found that apocynin-treated MSCs had better performance than naïve MSCs in the suppression of hematoma expansion, peri-hematoma neuronal death, and brain edema. Our results also suggest that the effect of apocynin-preconditioning on the therapeutic efficacy of MSCs may be related to the effect of MSCs on cerebrovascular integrity. Most importantly, this is the first study which suggests apocynin as a preconditioning regimen for enhancing the therapeutic efficacy of MSCs.

Early hematoma expansion is known as an important prognostic factor of ICH that can predict mortality and poor functional outcomes [12,26]. Various medical therapies have been attempted to overcome this poor prognosis, including the administration of corticosteroids, glycerol, and mannitol, however, none of them showed a beneficial effect in clinical trials [27,28,29]. Moreover, recombinant coagulation factor VII administration, which showed potential benefits in an early phase clinical trial, also failed to show a significant effect in a phase III clinical trial [30,31]. Hematoma size reduction is a well-known effect of MSC therapy for ICH, which was shown in previous research [32,33,34]. A recent preclinical study showed that MSCs administered at the acute stage of ICH could suppress early hematoma expansion through the enhancement of cerebrovascular integrity and decrease mortality [20]. Our study showed that apocynin augmented this effect of MSCs, and the finding supports the possibility of apocynin as a novel preconditioning regimen to enhance the therapeutic efficacy of MSCs and prevent the devastating consequences of early hematoma expansion. The result of western blotting showed the significantly higher expression of the tight junction protein occludin in the Apo-MSC group than in the naïve MSC and vehicle groups. ZO-1, another tight junction protein, also showed a similar trend of expression in each group, although there was no statistically significant difference between the Apo-MSC and naïve MSC groups. These results suggest that apocynin may enhance the effect of MSCs on endovascular integrity in cerebral vasculature and result in better performance in the suppression of early hematoma expansion. Furthermore, it is known that hematoma expansion occurs soon after onset in most cases of ICH, implying that there may be an active bleeding process in the acute phase of ICH [12,35]. Our results showed an improvement in the decrease of hematoma size and the increase of vascular integrity at the time point of 48 h after ICH induction in the Apo-MSC group as compared to that in the naïve MSC group. This suggests that apocynin preconditioning may enhance the efficacy of MSCs for stopping the process of active bleeding.

Hydrocephalus and cerebral edema are serious complications of ICH that cause an increase in intracranial pressure, therefore, most of the therapeutic strategies for the acute stage of ICH focus on preventing and treating these complications [14,18]. Our results showed a significant decrease in hemisphere enlargement in the Apo-MSC group as compared to that in the naïve MSC and vehicle groups, suggesting that apocynin preconditioning may enhance the therapeutic efficacy of MSCs in ICH complications, which was shown in the previous study [20]. Besides, the result of the FJC staining showed that apocynin-treated MSCs had better performance in decreasing perihematomal neuronal death than naïve MSCs. This finding suggests that apocynin may also augment the neuroprotective effect of MSCs. This may indicate that apocynin preconditioning could be used for enhancing the therapeutic efficacy of MSCs in various neurodegenerative diseases other than ICH. However, further study may be necessary to elucidate whether the result was a mere consequence of the enhanced effect in the decrease of hematoma expansion and intracranial pressure or due to the augmentation of the neuroprotective properties of MSCs due to apocynin. The finding might also result from apocynin released from Apo-MSCs, considering the effect of apocynin on neuronal injury shown in previous studies [36,37].

One of the most interesting findings of our study was the contradiction between the effect of apocynin on the therapeutic efficacy and the amount of exosome production in MSCs. Apocynin-preconditioned MSCs showed a greater efficacy than naïve MSCs despite showing a decrease in exosome production. This finding is contradictory considering that the exosome is currently believed to be one of the major routes through which MSCs show their therapeutic action [38]. The decrease in exosome production shown in our results was rather consistent with the concept of hypoxia preconditioning which is generally expected to activate NADPH oxidase in contrast to apocynin [21,22,25]. This contradiction in the effect on NADPH oxidase activity between hypoxia (one of the conventional preconditioning methods) and apocynin preconditioning suggests that the underlying mechanism of both hypoxia and apocynin preconditioning may not be related to NADPH oxidase activity itself. The reversal of the aging process in MSCs by apocynin, which was shown in a prior study, may be another possibility that explains the effect of apocynin preconditioning on the therapeutic efficacy of MSCs in ICH [24]. Alternatively, this decrease in exosome production might merely reflect the prolonged cell doubling time. Further study may be required to clearly elucidate the mechanism underlying this effect of apocynin preconditioning.

In conclusion, apocynin treatment enhances the therapeutic efficacy of MSCs in ICH in the acute stage through the improvement of the beneficial properties of MSCs, such as neuroprotection and the reinforcement of endovascular integrity in cerebral vasculature.

Our study has a few limitations. First, we did not suggest a clear molecular mechanism underlying the effect of apocynin-preconditioning on the therapeutic effect of MSCs for ICH. This may limit the possibility of apocynin as a novel preconditioning regimen to enhance the therapeutic properties of MSCs. However, the prominent and consistent differences between the Apo-MSC and naïve MSC groups shown in most tests increase this possibility. Further study to confirm the effect and elucidate the underlying mechanism should be carried out. Secondly, we did not clearly confirm which property of apocynin enhances the efficacy of MSCs in ICH. We assumed that the change in the characteristics of MSCs might be related with NADPH oxidase inhibition based on the results of prior research [24]. This should also be clarified in future studies. Thirdly, we only assessed the effect of a single dose administration of MSCs. Repeated doses may affect the therapeutic efficacy of MSCs on ICH, considering the result of prior research which showed that the repeated administration of MSCs affected the endogenous neurogenesis activity after global cerebral ischemia [39].

## 4. Materials and Methods

### 4.1. Experimental Animals

Animal care protocol and experimental procedures were approved by the Institutional Animal Care and Use Committees (IACUC no: 180065, approval date: April 9, 2018). All experiments were performed according to relevant guidelines and regulations. Adult male Sprague-Dawley rats (8-week-old, weighing 250–350 g) were used in this study. The rats were housed in a regulated environment (22 ± 2 °C, 55 ± 5% humidity, 12 h light/dark cycles) with free access to food and water. 

### 4.2. MSC Preparation and Apocynin Preconditioning

The hPDMSCs (CHA Biotech, Seongnam, Korea) were maintained as previously described [20]. Briefly, the hPDMSCs were cultured in MEM-α GlutaMAX (Gibco, Grand island, NY, USA) which was supplemented with 10% fetal bovine serum (Biowest, Riverside, MO, USA), 20 ng/mL basic fibroblast growth factor (bFGF) (PeproTech, Rocky Hill, NJ, USA), and 50 μg/mL gentamycin (Sigma-Aldrich, St. Louis, MO, USA) at 37 °C in an atmosphere of 5% CO_2_. Apocynin (EMD Millipore, Billerica, MA, USA) was dissolved in dimethyl sulfoxide (DMSO). In order to produce five doses of apocynin-preconditioned placenta-derived MSCs, 2.5 × 10^6^ cells at passage 6 were treated with 100 μM of apocynin (total media volume 35 mL) and incubated for 72 h. All experiments were performed at passage 7.

### 4.3. ICH Model

**Figure 4:**
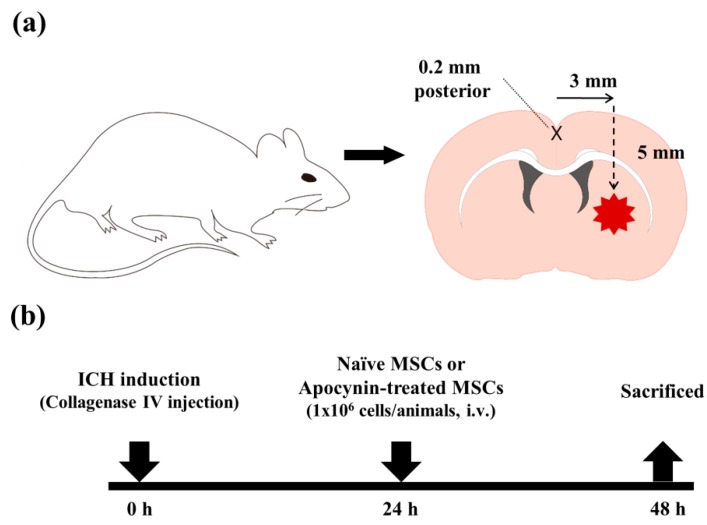
Conceptual illustrations of the experimental protocol. (**a**) Schematic diagrams of a rat intracranial hemorrhage (ICH) model. ICH was induced by the infusion of bacterial collagenase type IV (0.1 U, 1 μL) into the striatum. (**b**) Brief timeline of the experimental procedures.

To reproduce an ICH with ongoing bleeding we injected bacterial collagenase intrastriatally as previously described [40]. Rats were deeply anesthetized with ketamine and xylazine and placed in a stereotaxic frame (Kopf Instruments, Tujunga, CA, USA). A burr hole was made and a 26-gauge needle was inserted through the burr hole into the striatum (coordinates: 0.2 mm posterior, 5.0 mm ventral, and 3.0 mm lateral to bregma, Figure 4a). We then injected collagenase type IV (0.1 IU/μL) for 5 min. After placement for another 4 min, the needle was removed in 2 min. The burr hole was sealed with bone wax. Following suturing of the skin incision, the core temperature was kept at 35.5–36.5 °C with a homoeothermic control (LMS Korea, Seongnam, Korea). Sham-operated rats received the same skin incision under ketamine and xylazine anesthesia but they were administered 1 μL of sterile saline into the right striatum. Rats were anesthetized by ketamine and xylazine and the administration of MSCs was performed via the femoral vein at 24 h after ICH induction (Figure 4b).

### 4.4. Tissue Preparation

At 24 h after cell injection (48 h after ICH induction), the rats were anesthetized and transcardially perfused with heparinized saline followed by 4% paraformaldehyde (PFA; EMD Millipore, Billerica, MA, USA) in saline. The brains were post-fixed with 4% PFA overnight at 4 °C and then immersed in 30% sucrose for cryoprotection. The rat brains were embedded in a frozen section embedding medium (Leica, Richmond, IL, USA) and stored at −80 °C. Thereafter, the entire brain was frozen and coronally sectioned with a cryostat microtome at 30-μm thickness. 

### 4.5. Measurement of the Hematoma Volume and Hemispheric Enlargement

The hematoma volume was quantified using coronal sections at 28 rostral-caudal levels that were spaced every 1 mm from +1.20 mm to −3.80 mm relative to the bregma. The volume measurement was computed by the summation of the areas multiplied by the interslice distance (270 μm). Digital photographs of the serial slices were taken and the percentage of hematoma volume (hematoma volume/hemispheric brain volume × 100) was measured using the Image J (NIH, Bethesda, MA, USA) software [41].

Brain edema was also measured using the Image J software as the percentage of hemispheric enlargement, which was calculated by the following formula: (Ipsilateral hemisphere volume – contralateral hemisphere volume)/contralateral hemisphere volume × 100.

### 4.6. Detection of Neuronal Death

Neuronal death was evaluated by Fluoro-Jade C (FJC, EMD Millipore, Billerica, MA, USA) staining 48 h after ICH. The sections were rinsed in phosphate-buffered saline (PBS), mounted onto gelatin-coated slides, and dried on a slide warmer. The slides were immersed in 80% ethanol for 5 min, followed by 70% ethanol for 2 min and distilled water (DW) for 2 min. The slides were then transferred to 0.06% potassium permanganate for 10 min and gently agitated. After rinsing in DW for 1 min, the slides were incubated for 10 min in 0.001% FJC dissolved in 0.1% acetic acid. After rinsing for 1 min in each of the three changes of DW, the slides were air dried then dehydrated in xylene for at least 1 min and coverslipped with DPX (Sigma-Aldrich Co., St. Louis, MO, USA). To quantify neuronal death in the subventricular zone (SVZ) after ICH, SVZ sections were analyzed from each animal using an objective microscope with a 20× magnification. An observer masked to the treatment condition counted the number of FJC-positive (+) neurons by sampling an area of 471.87 × 471.87 μm^2^ immediately adjacent to the hematoma in four regions of interest (ROIs) from the ipsilateral hemisphere after the induction of the ICH. The mean numbers of FJC (+) neurons from the four ROIs were used for statistical analyses.

### 4.7. Western Blotting

Rats were anesthetized and decapitated 48 h after ICH or sham operation for the analysis of the expression of tight junction proteins using western blotting. A 3 mm coronal section from the ipsilateral hemisphere was homogenized in an ice-cold RIPA buffer (Thermo Fisher Scientific, Waltham, MA, USA). The total protein concentration was measured using a bicinchoninic acid (BCA) Protein Assay Kit (Thermo Fisher Scientific, Waltham, MA, USA). Then, 20 μg of protein from each sample was subjected to sodium dodecyl sulfate–polyacrylamide gel electrophoresis (SDS-PAGE) and transferred to a polyvinylidene difluoride (PVDF) membrane (GE Healthcare Bio-Sciences, Pittsburgh, PA, USA). The membranes were then probed with the following primary antibodies: Rabbit anti-occludin (Thermo Fisher Scientific, Waltham, MA, USA), rabbit anti-zonula occludens-1 (ZO-1) (Abcam, Cambridge, MA, USA), and mouse anti-β-actin (Santa Cruz Biotechnology Inc., Dallas, TX, USA). β-actin was used as an internal loading control. The secondary antibodies were all acquired from GeneTex (Irvine, CA, USA). Western blotting was performed with an ECL Detection Kit (Bio-rad, Hercules, CA, USA). The relative band density of each sample was analyzed using the Image J software (https://imagej.nih.gov/ij/).

### 4.8. Exosome Isolation and Quantification

To isolate exosomes from the cell culture medium, naïve MSCs and apocynin-treated MSCs were grown in an exosome-depleted culture medium with or without apocynin (100 μM). The exosome-depleted culture medium was composed of MEM-α GlutaMAX (Gibco, Grand island, NY, USA), supplemented with 10% exosome-depleted fetal bovine serum (SBI, Palo Alto, CA, USA), 50 μg/mL gentamycin (Sigma-Aldrich, St. Louis, MO, USA) and 20 ng/mL bFGF (PeproTech, Rocky Hill, NJ, USA). After 72 h of incubation, the concentrated culture medium was collected and centrifuged at 3000× *g* for 10 min to discard cell debris. The supernatant was harvested and ultracentrifuged at 100,000× *g* for 90 min to isolate the exosomes. The pellets were washed with Dulbecco’s phosphate-buffered saline (DPBS) and ultracentrifuged again at 100,000× *g* for 90 min to collect the exosomes. The isolated exosome pellets were resuspended with DPBS containing 10% glycerol. Exosomes derived from 200 cc of the culture medium were quantified using the bicinchoninic acid (BCA) protein assay [42].

### 4.9. Statistical Analysis

Comparisons between the Apo-MSC, naïve MSC and vehicle groups were performed using the t-test. Data are presented as the mean ± SD and differences were considered significant at *p* < 0.05. Microsoft Excel 2016 (Microsoft, Redmond, WA, USA) was used for data presentation and statistical calculation. A sample size of 15 was calculated to detect a significant difference in the hematoma size at 48 h post-ICH induction among the Apo-MSC, naïve MSC and vehicle groups (power = 0.95), using G*Power 3.1 (Heinlich-Heine Universität, Düsseldorf, Germany) [43].
